# A randomized controlled trial of a psychoeducational group program for unipolar depression in adults in Norway (NCT00319540)

**DOI:** 10.1186/1745-0179-2-15

**Published:** 2006-06-28

**Authors:** Odd Steffen Dalgard

**Affiliations:** 1 Norwegian Institute of Public Health, Marcus Thranes gt. 6, P.O. 4404 Oslo, Norway

## Abstract

**Background:**

Coping with Depression Course (CWD) has shown to be effective in the treatment of depression. However, there are very few randomized controlled trials on unipolar depression in adults

**Aims:**

To test the effect of a modified CWD on unipolar depression in a randomized controlled trial design in adults.

**Methods:**

Participants were recruited through mass media, tested by BDI and clinical interview, and randomized into intervention group (N = 81) and control group (N = 74). The program was mainly conducted by nurses with background in psychiatry and primary health care, and the intervention encompassed 8 weekly sessions of 2 1/2 hours, with 3 booster sessions.

**Results:**

By "intention-to-treat" analysis a statistically significant effect on depressive symptoms at follow up at 6 months was found, and the level of symptoms was sustained after 12 months.

**Conclusion:**

The study shows that the intervention is effective in the treatment of unipolar depression, and suitable for specialized psychiatric services as well as primary health care.

## Background

Various types of psychotherapy have shown to be effective in the treatment of depression. Compared to treatment with antidepressants, the effect seems to be on the same level, at least in moderate and mild depression [1], and this applies to different types of psychotherapy [2-4]. With respect to type of staff, there seems to be no major difference in results between highly specialised and less specialised staff when dealing with group treatment [5].

The Coping with Depression Course (CWD), a psycho-educational group program developed by Lewinsohn and coworkers [6,7], has been widely used to treat unipolar depression [8]. A recent meta-analysis [9], summarising 24 studies, shows that CWD, and its variants, is effective in reducing symptoms of depression. Only four of these studies, however, are randomised controlled trials on clinical depression [10,6,12], and of these only Brown *et al *and Dowrick *et al *are dealing with adults.

The study of Dowrick *et al*, the so called ODIN study (Outcome of Depression in Europe), is a multicite study of CWD, as modified by Munoz [13], and carried out within the frame of an epidemiological survey [14]. The study showed effect after 6 months, but after 12 months there was no difference between intervention group and control group. Especially in Norway, the rate of refusal was high (28/78), which made it difficult to assess the effect. The high refusal rate may be explained by the design of the study, where people who had not searched for treatment themselves were invited to participate.

To overcome the problem of high refusal rate and a relatively small sample, the aim of the present study was to test a slightly modified version of the ODIN model in a naturalistic setting in Oslo, with self-referred and motivated participants.

## Methods

### Sample

The candidates for the study were recruited through advertisement in an Oslo newspaper, with a short description of the course. The assessment of applicants took place in the office of the Norwegian Council for Mental Health in Oslo.

The eligibility criteria was unipolar depression according to DSM IV. The assessment was done by interviewing by a psychiatrist or a clinical psychologist on the basis of a semi-structured interview. Both interviewers were recently trained in the use of SCAN, 2.0, and the diagnostic assessment was based on principles of SCAN. People with psychotic symptoms, other psychiatric diagnosis than unipolar depression, suicidal ideation or obvious learning disabilities were not accepted.

In a few days, more than 300 took contact by telephone, and of those the first 182 were invited for an interview. Of these, 155 were found suitable for the course. The reasons for not accepting applicants were: psychosis (4), subclinical depression (12), other psychiatric diagnosis (3), risk of suicide (1), preference of other therapy (2), lack of cognitive skills (4), other reasons (1).

### The intervention

The course consisted of 8 weekly sessions, each of 2,5 hours duration, and booster sessions 1, 2 and 4 months after the course. Each group counted 8–10 participants, was lead by two professionals, mainly nurses with background in psychiatry and primary health services, and took place in primary health clinics. The group leaders had completed a training course in 3 phases: Theoretical background, own participation in course and conducting of course under supervision. The present study is based on the last of these phases, which means that it is an evaluation of a course led by people in a training situation.

The course emphasized teaching and not therapy, and aimed at promoting positive thinking, pleasant activities, social skills and social support. The last component, with drawing of network map and stimulation of participant contacts between the sessions, made the course somewhat different from courses exclusively based on cognitive/educational principles. Home work was an important part of the course, and written hand- outs were used extensively. Compared to CWD in the ODIN study, the handouts were considerably enlarged, with more theory and more examples. The booster sessions, which were not part of the ODIN study, aimed at repetition of the main points of the course, but were otherwise open for input from the participants themselves.

The control group as well as the intervention group were free to continue eventual ongoing treatment (i.e. "treatment as usual"). As shown in table 1, 44,4% of the intervention group and 42.7% of the control group were on medication at program start and respectively 24.0% and 12.0% on psychotherapy. To which extent this treatment was continued during the program, is, however, not known.

**Table 1:** The sample. For categorical variables: Percent, absolute numbers in brackets. For continues variables: Mean, SD in brackets

		Intervention	Control	P value
Gender	Men	24.7 (20)	23,0 (17)	0.476
	Women	75.3 (61)	77,0 (57)	
Age	Mean	44.5 (10.8)	50.3 (11.9)	0.002
Marital status	Unmarried	30.0 (24)	14.9 (11)	0.107.
	Married/cohabitant	45.0 (36)	54.1 (40)	
	Divorced/separated	22,5 (18)	24.3 (18)	
	Widow/widover	2.5 (2)	6.8 (5)	
Education	7 years	6,2 (5)	5.4 (4)	0.369
	8– 10 years	11.1 (9)	17.6 (13)	
	11 – 12 years	16.0 (13)	23.0 (17)	
	13 years and more	66.7 (54)	54.1 (40)	
Duration of depression	>2 years	53.1 (43)	62.0 (44)	0.174.
BDI	Mean	21.8 (7.9)	22.9 (8.2)	0.507
Medication	Now	44.4 (36)	42.7 (32)	0.475
	Earlier	16.0 (13)	24.0 (18)	0.149
Psychotherapy	Now	24.0 (19)	12.0 (9)	0.048
	Earlier	44.4 (36)	34.7 (26)	0.139

### Design

The study encompasses two waves of interventions. Only the *first wave *is a randomized controlled trial, with comparable follow up data at 6 months after program start for intervention group and controls. The *second wave *of interventions was offered to those who were controls in the first wave, and started about 6 months after the initial program start. This group was followed up 6 months later, with no control group.

### Outcome measures

The outcome measure was change in scores on Beck Depression Inventory (BDI). This was estimated in three different ways, considering BDI change of 6 BDI points as reliable and clinically interesting [5], and BDI 10 as cut off point for depression:

• Proportion improved 6 BDI points or more at follow up.

• Proportion with BDI<10 at follow up.

• Mean differences in BDI scores from start to follow up.

### Sample size

The sample size (N = 155) was determined by practical restrictions and estimation of statistical power. The statistical power for detecting a change of 6 BDI points or more at 6 months follow up with p < 0.01, was 99%.

### Randomization

Every second person on a list of names (N = 155) was assigned to the intervention group, the others to the control group. The sequence of names on the list was ordered according to time of recruitment. Randomization and assignment to groups was done by a research assistant. For the purpose of having about 8 participants in each of 10 intervention groups, 3 persons were moved from the control group to the intervention group. These persons were randomly drawn from the list of controls.

The follow up data were collected by postal questionnaires, and those scoring the outcomes were blinded to group assignment.

### Statistical methods

In comparing binary outcomes chi-quadrate test was used, and in comparing differences in means analysis of variance (One-Way ANOVA) was used. In multivariate analyses with binary outcome logistic regression was used, and with continuous outcome linear regression.

P-values were estimated for all comparisons, and 95% Confidence Intervals were estimated for logistic regression.

## Results

First wave intervention

### Participant flow

**Figure 1:**
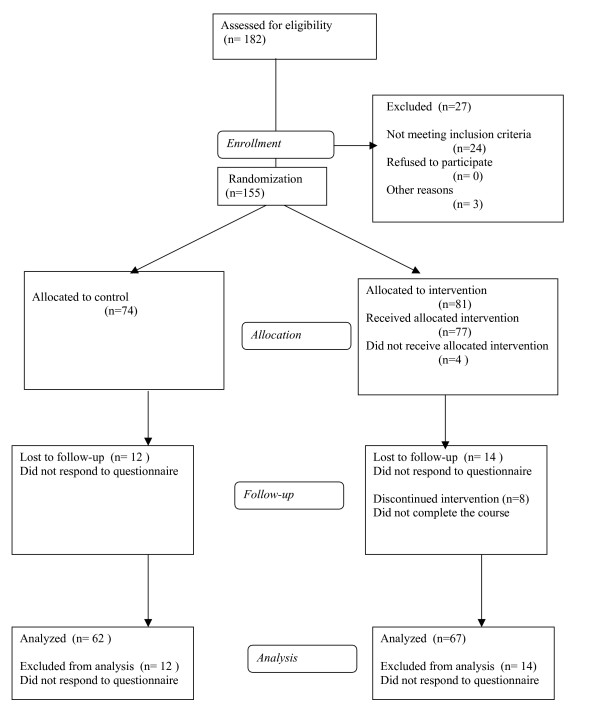
Flow chart of participants.

The flow of participants is shown in figure 1.

Of those allocated for intervention, 4 persons refused to participate, and 8 persons did not complete the course. This means that 85% of those allocated to intervention completed the course.

Of those allocated for intervention, 83% took part in the follow up, whereas the corresponding number of controls was 82%. Those lost to follow-up did not respond to the postal questionnaire.

### Recruitment

The recruitment of candidates was done by advertisement in a major newspaper in Oslo in January 2001. The first wave of interventions started in March/April 2001, about 4 weeks after the interview of candidates. The group participants were subject to follow up 2 months after program start, whereas all allocated for intervention or control group were subject to follow-up after 6 months. A last follow up of the intervention group was done in March/April 2002, 12 months after program start. The second wave of interventions, for the prior controls, started in September 2001 with follow up 6 months later. The intervention was administered in primary health centres.

### Baseline data

The baseline data are shown in table 1.

The sample is clearly different from an average Oslo population, with a higher proportion of women, divorced/separated and people with higher education. With respect to depression, the level of BDI indicates a moderately depressed group, with a mean score of about 22. A few had a BDI score less than 10, five participants and 4 controls, which could indicate that they were not depressed. The clinical assessment, however, concluded with unipolar depression. The mean duration of depression was relatively high, with more than 50% having been depressed for more than two years. It was characteristic for the group that a high percentage was, or had been in psychotherapy, and used or had used medication. In total, 25% used SSRIs, 5% tricyclic antidepressants and 14% sedatives or anxiolytics. There were small differences between the intervention group and controls, and only for age and psychotherapy the differences were significant. The intervention group was somewhat younger, and more often received psychotherapy at the start of intervention.

At the follow up at 6 months the sample was subject to "intention-to-treat analyses", meaning that all persons allocated to intervention were included in the analysis, irrespective of actual participation in the intervention or not. The only group not included in the analyses was those who did not respond to the follow up questionnaire (N = 26). This group did not differ significantly from the others with respect to baseline BDI, duration of depression at baseline, or the main background variables which might influence the course of depression.

### Outcomes and estimation

The proportion with improvement of 6 BDI points or more, was 69% (46/67) in the intervention group and 37% (23/62) in the control group. The % difference, 32, was statistically significant (p < 0.001, 95%CI 15–48). The proportion with with BDI<10 points after 6 months was 36% (23/64) in the intervention group and 20% (12/59) in the control group. The % difference, 16, was not statistically significant (p = 0.08, 95% CI -2-33). However, it indicates that the number needed to treat (NNT) to "cure" one person for depression (i.e. reducing BDI below 10) was about 6. The results of multivariate analyses, when adjusting for socio-demographic factors, duration of depression, treatment at baseline and level of depression, all at baseline, are shown in table 2.

**Table 2:** Logistic regression analysis

Dependent variable: BDI reduction > 6 points			
	OR	95%CI	Significance

Gender	1.65	0.57–4.75	0.356
Age	0.99	0.95–1.03	0.486
Marital status	0.88	0.38–2.06	0.783
Education	0.91	0.58–1.44	0.687
Duration of depression	0.26	0.10–0.63	0.003
Medication baseline	0.84	0.61–1.15	0.279
Psychotherapy baseline	3.35	1.04–10.78	0.043
BDI baseline	1.07	1.01–1.13	0.019
**Control/Intervention**	**3.42**	**1.47–7.96**	**0.004**

Dependent variable: BDI<10 at 6 month follow up*.			

	OR	95%CI	Significance p-values

Gender	1.40	0.43–4.50	0.577
Age	1.00	0.96–1.05	0.876
Marital status	0.84	0.33–2.18	0.726
Education	1.04	0.63–1.72	0.870
Duration of depression	0.20	0.07–0.49	0.001
Medication baseline	0.95	0.68–1.33	0.772
Psychotherapy baseline	0.62	0.18–2.14	0.447
BDI baseline	0.88	0.82–0.95	0.000
**Control/Intervention**	**2.50**	**0.95–6.56**	**0.063**

* Those with BDI<10 at program start are excluded

The effect of intervention is statistically significant (OR 3.42, p = 0.004) when reduction of BDI more than 6 months was used as outcome. Duration of depression at baseline has a negative effect on outcome, whereas level of BDI and psychotherapy at baseline has a positive effect. The effect of intervention is close to statistically significant (2.50, p = 0.063) when BDI <10 at follow up was used as outcome. Duration of depression and BDI at baseline have a negative effect also on this outcome measure.

The mean differences in BDI scores between program start and 6 months follow up are 8.25 (SD 8.16) in the intervention group and 4.42 (SD 8.30) in the control group. The difference between the two groups is statistically significant (p = 0.009).*Cohens d*, (*mean *intervention group – *mean *controls/SD) was 0.47.

The result of multivariate analysis, when adjusting for the same variables as in table 2, is shown in table 3.

**Table 3:** Multiple linear regression analysis

Dependent variable: BDI difference		
	Standardized beta Coefficients	Significance p-values

Gender	0.052	0.540
Age	-0.048	0.588
Marital status	-0.034	0.683
Education	-0.001	0.987
Duration of depression	-0.327	<0.001
Medication baseline	-0.009	0.913
Psychotherapy baseline	0.151	0.075
BDI baseline	0.336	<0.001
**Control/Intervention**	**0.184**	**0.030**

The effect of intervention is statistically significant (p = 0.030). The duration of depression at baseline has a negative effect (p < 0.001), whereas BDI at baseline has a positive effect (p < 0.001).

The main effect of the intervention seemed to appear after the 8 group sessions, and before the booster sessions. During these two first months the mean BDI difference in the intervention group was 6.90 (SD 7.43) against 8.25 (SD 8.16) at 6 months. However, there was no comparable data for the control group.

At follow up after 12 months the BDI of the intervention group remained quite stable, the mean difference being 7.93.

#### Second wave intervention

The initial controls were offered an intervention course 6 months after program start, and 16 persons accepted this offer (i.e. two intervention groups). When comparing the BDI score of this intervention group at the start of the second wave, with their score at program start, there was no difference (the mean score at both points in time being 22.88). This means that this group had shown less improvement than the total control group of the first wave, where the mean reduction in BDI score was 4.42.

During the second wave intervention 56% had reduced their BDI score 6 points or more, and 28% had a BDI score less than 10 at 6 months follow up. The corresponding figures for the first wave intervention being 69% and 36% respectively, indicate that the improvement in the second wave intervention was less than in the first. The mean BDI difference score was also less in the second wave than in first, the figures being 4.00 and 8.25 respectively.

## Discussion

### Interpretation

It is a strength of the present study that it is based on a randomized controlled trial with a relatively great sample, and addresses an important health problem where such studies are rare. The problem of depression seems to be growing on a global scale [15], and represents a great challenge to the health services, not least to the primary health services.

From a practical point of view, it is an advantage that the study deals with a group- based intervention, feasible also in the primary health services, without the need of highly specialized staff, like psychiatrists or clinical psychologists. From a research point of view it is positive that the attrition rate is low, and that the people lost to follow up are not different from the rest with respect to background factors. This allows for drawing firm conclusions from the sample.

On the other side, it is a weakness that diagnostic interviews are conducted only at the start of the program, and even if the interviews were based on the principle of SCAN, it is a weakness of the study that the interviews did not strictly follow the rules of this instrument. It is also a weakness that the study employed only a single outcome measure, Beck Depression Inventory. Further studies should include more outcome data, like data on social adjustment and quality of life (such data was actually included in the present study, but invalidated because of technical reasons). It is also a weakness that the follow up in the randomized trial was limited to a relatively short time span, i.e. 6 month.

The effect of the intervention is at about the same level as reported from other studies of psychotherapy of unipolar depression [3-5], and *Cohens d *at 0.47 indicates a moderate effect. In the metastudy of CWD and its variants referred to in the introduction [9], the mean *Cohens d *was 0.47. Compared to CWD in the ODIN study [12], the effect of the intervention seems slightly better, the NNT being respectively 6 and 7. This is in accordance with other studies [9], reporting that self-referred clients, as in the present case, display larger improvement effects than study-referred clients. The low compliance rate in the ODIN study could also contribute to a less positive outcome in an "intention- to- treat" design. When the results are not as good in the second wave intervention as in the first, this may be explained by the participants in the second wave having shown a stronger tendency towards chronicity even before the intervention.

With respect to eventual modifications of the course on the bases of the present study, one may question if the booster sessions are needed, as nearly all improvement seemed to take place before these sessions. However, it is also possible that the booster sessions contributed to the prevention of relapse after the first 2 months.

As the course had cognitive and behavioural components, as well as a component of social support, one may ask what was most important. The present study does not allow for singling out partial effects, as the different components are closely integrated. The relatively strong focus on social support is in line with the emphasizing of social contacts in the theoretical part of the course. Anyhow, it makes the present course somewhat different from courses which focus more exclusively on the cognitive/behavioural components.

### Generalizability

Even if the course has demonstrated significant effect on unipolar depression in the present sample, one may questions to which extent the results can be generalized to depressed patients in general. As people with psychosis, bipolar affective disorder, subclinical depression, suicidal thoughts and obvious learning disability were left out, the study cannot say anything about the effect on these groups. The overrepresentation of people with higher education may indicate that the course is most suitable for people with cognitive skills above the average, but this should be subject for further research. Taken into consideration that the participants were strongly motivated for a course like the present one, since they to a great extent had experienced other types of psychotherapy and medication without satisfactory results, the participants obviously represent a special selection. This selection, however, should not necessarily favour a positive outcome, since it consists of a group with long duration of illness (58% having been depressed more than two years), and with little effect of other types of treatment. It is likely that the course would be even more effective in people with depression of shorter duration. This assumption is supported by the negative effect on outcome of duration of depression in the logistic regression analysis.

When assessing the effect of the program, one also has to take into consideration the level of competency of the group leaders. In the present study, the intervention was part of a training program of group leaders, which means that they had no prior experience from conducting courses like this. This makes it likely that the effect will be even better with more experienced group leaders.

As mentioned in the introduction, the CWD course in the ODIN project had a rather low acceptability, probably because of fear of stigmatization. The present study, dealing with a special selection of highly motivated people, does not directly address the question of acceptability in the general population. However, after the training of more than 200 group leaders, mainly psychiatric nurses, the course has now been implemented in more than 2000 depressed patients in Norway, mainly referred from General Practitioners. Fear of stigmatization doesn't seem to be a problem in this setting, where people themselves are searching for help, as contrary to the situation in the ODIN project, where depressed people were identified by an epidemiological study.

### Overall evidence

The study indicates that the intervention is effective in people with unipolar depression with relatively long duration of illness, where other types of treatment have failed or had a limited effect. The course is especially suitable for the primary health services, and does not require highly specialized staff, like psychiatrists or clinical psychologists.

## Competing interests

The author has shared ownership in firm editing printed material for the course.
